# Development of a quadruplex RT-qPCR for the detection of avian leukosis virus, chicken infectious anemia virus, avian reovirus, and fowl adenovirus

**DOI:** 10.3389/fvets.2026.1747413

**Published:** 2026-03-18

**Authors:** Haozhao Mo, Kaichuang Shi, Yu Gan, Yanwen Yin, Feng Long, Shuping Feng, Sujie Qu, Wenjun Lu, Xingju Song

**Affiliations:** 1 College of Animal Science and Technology, Guangxi University, Nanning, China; 2 Guangxi Center for Animal Disease Control and Prevention, Nanning, China

**Keywords:** avian leukosis virus, chicken infectious anemia virus, avian reovirus, fowl adenovirus, detection method, multiplex RT-qPCR

## Abstract

Avian leukosis virus (ALV), chicken infectious anemia virus (CIAV), avian reovirus (ARV), and fowl adenovirus (FAdV) are important viral pathogens that can transmitted horizontally and vertically, and induce immunosuppression to the poultry flocks. They exhibit diverse pathogenic characteristics in clinical settings, and pose continuous threat to the health of poultry flocks. Here, the specific primers and probes were designed for the env gene of ALV, the VP1 gene of CIAV, the M1 gene of ARV, and the ORF1 gene of FAdV. The RNA standards for ALV, and ARV, and the plasmid DNA standards for CIAV, and FAdV were constructed. To establish a quadruplex real-time quantitative PCR (RT-qPCR) for detecting these four viruses, the reaction conditions (primer and probe concentrations, annealing temperature, and reaction cycles) were optimized, and the specificity, sensitivity, and repeatability were analyzed. The results indicated that the developed assay could specifically detect ALV, CIAV, ARV, and FAdV, and had no cross-reaction with other chicken viruses; the limits of detection (LODs) of them were 136.66, 129.59, 133.20, and 139.79 copies/reaction, respectively, demonstrating high specificity and sensitivity. In addition, this assay had excellent repeatability, with coefficients of variation (CVs) of 0.29–0.93% for the intra-assay and of 0.29–0.99% for the inter-assay. The developed assay was validated via testing 1,575 clinical samples from Guangxi province, China. The positivity rates of ALV, CIAV, ARV, and FAdV were 36.89% (581/1,575), 17.65% (278/1,575), 2.16% (34/1,575), and 7.05% (111/1,575), respectively. These 1,575 clinical samples were also tested using the reported reference methods, and the results were compared with those of the established method. The coincidence rate of the developed and the reference assays exceeded 99.31%. In conclusion, a quadruplex RT-qPCR was successfully developed for the efficient and precise detection and differentiation of ALV, CIAV, ARV, and FAdV.

## 1 Introduction

Avian leukosis virus (ALV), which belongs to the genus *Alpha-retrovirus* in the *Retroviridae* family, is a single-stranded, positive sense RNA virus with a 7.8 kb genome in the arrangement order of 5′ LTR-gag/pro-pol-env-LTR 3′ (1). The env gene is related to pathogenicity, and there are significant differences in this gene among different ALV subtypes. The Env protein consists of gp85 and gp37 subunits (1). The gp85 protein constitutes the surface portion of the viral structural proteins, and is closely related to the process of the viral binding to cell receptors and the determination of subtype specificity (1). ALV is classified into 11 subtypes (A-K) (2), and J subtype (ALV-J) is the predominant subtype in different countries nowadays (1). ALV-J was first isolated from commercial broilers in the United Kingdom (UK) in 1988. The first case of ALV infection in white-feathered broilers was first confirmed in China in 1999 (2), and ALV has been reported in many provinces in China (2–7). As an oncogenic exogenous retrovirus, ALV is a virus produced by the recombination of endogenous and exogenous viruses, which causes malignant tumors such as myeloid leukemia, hemangioma, and renal tumor (1–2).

Chicken infectious anemia virus (CIAV), which belongs to the genus *Gyrovirus* in the family *Anelloviridae*, is a non-enveloped, single-stranded DNA virus with a 2.3 kb genome (8). The viral genome has three partially or completely overlapping open reading frames (ORFs) in the same direction, encoding the VP1 (1.3 kb, 51.6 kDa), VP2 (651 bp, 24 kDa), and VP3 (366 bp, 13.6 kDa) proteins, respectively. The ORF of VP2 is completely overlapped with that of VP3, and partially overlapped with that of VP1 (8). Chickens are the main natural hosts of CIAV, and chickens of all ages are susceptible to CIAV (8). CIAV was first reported in Japan in 1979, and at present is widespread worldwide (9). CIAV has been reported in many provinces in China since 1996 (10–12). The main characteristic of CIAV infection is anemia, which leads to a decrease in the hematocrit of infected chickens. In severe cases, the infected chickens might show clinical manifestations such as diarrhea, subcutaneous hemorrhage and edema (8, 9).

Avian reovirus (ARV), which belongs to the genus *Orthoreovirus* in the *Reoviridae* family, is a non-enveloped, double-stranded RNA virus with a 2.3 kb genome (13). The genomic fragments are classified into three size categories: L-class, M-class, and S-class, and are further subdivided into subcategories: L1, L2, L3, M1, M2, M3, S1, S2, S3, and S4 (13). The σC protein encoded by S-class genes has the function of evading cellular immunity, so this protein is the most prone to mutation in the genome (13). Based on the σC gene locus, ARV can be classified into six genotypes, and different genotypes might have differences in pathogenicity (14, 15). ARV was first reported in South Africa in 1950, and has been prevalent in different countries around the world (13, 16). ARV was first reported in China in 1986, and has been reported in many provinces throughout the country (14, 17–19). Chickens infected with different ARV strains might show stunted growth, hepatitis, myocarditis, tenosynovitis, viral arthritis and malabsorption syndrome (13–15).

Fowl adenovirus (FAdV), which belongs to the genus *Aviadenovirus* in the family *Aviadenoviridae*, is a non-enveloped, single-linear, double-stranded DNA virus with a 2.8–4.5 kb genome (20). The genomic structure is divided into early transcription units (E), late transcription units (L), and inverted terminal repeats (ITR). E zone expressed proteins are involved in the transcription of viral genes, and they are the initiating factors for viral gene activity. L region consists of L1-L5, and its gene products are the main structural proteins of the virus (21). The genus *Aviadenovirus* is subdivided into five species (A, B, C, D, and E) and twelve serotypes (1 to 11, among which type 8 is further divided into 8a and 8b). FAdV was first reported in Pakistan in 1987, and has been reported in many provinces in China (22–24). The coexistence of multiple serotypes makes the prevention and control of avian adenoviruses more complicated. E-8a, E-8b, and D-11 serotypes can induce inclusion body hepatitis (IBH) (25). Adenoviruses of different serotypes have distinct pathogenicity and clinical manifestations. Chickens infected with FAdV show symptoms such as listlessness, slow growth, decreased egg production, liver damage and anemia (20, 25).

ALV, CIAV, ARV, and FAdV are highly infectious pathogens that commonly affect poultry industry worldwide. These viruses can spread through horizonal and vertical transmission by different routes such as eggs, feces, and aerosols, which makes them difficult to eradicate once established in poultry flocks. What’s worse, they induce immunosuppression through distinct mechanisms: ALV infects lymphocytes and macrophages, impairing cellular and humoral immunity (3); CIAV attacks hematopoietic stem cells and thymic cells, causing severe immunodeficiency (8); ARV targets bursal and splenic lymphocytes, reducing antibody production (15); FAdV induces lymphocyte apoptosis in the thymus and bursa, leading to transient immunosuppression (25). They cause characteristic, and also similar clinical symptoms. For example, they greatly reduce the growth and development of chicks, the production efficiency of laying hens and broiler breeders, and aplastic anemia and enlarged blood vessels and organs in chickens (1, 2, 13–15, 20, 25). Furthermore, ALV, CIAV, ARV, and FAdV are prone to co-infection, which leads to serious immunosuppression and makes the animals vulnerable to invasion by other pathogens, thereby exacerbating the clinical symptoms and pathological changes (26–29). Especially, in the early stage of infection or the subclinical infections in the poultry flocks, the clinical symptoms and pathological changes of these four viruses are difficult to differentiate, and the differential diagnosis is hard to made depending only on the clinical signs and pathological changes, especially the co-infected cases. It is necessary to detect the pathogens relying on laboratory testing techniques, of which the RT-qPCR is an important detection method. RT-qPCR can precisely amplify the nucleic acid sequence of pathogens through the design of specific primers, and effectively detect the samples with extremely low concentrations of templates (30). Especially, the multiplex RT-qPCR can detect several pathogens in one reaction, with highly cheap, specific, sensitive, and accurate (31, 32). At present, there are reports on the RT-qPCR methods for the individual detection of ALV, CIAV, ARV, or FAdV (33–37). These singleplex assays could specifically detect ALV, CIAV, ARV, or FAdV with high specificity and sensitivity, respectively. In addition, a triplex RT-qPCR for the detection of FAdV type I, type III, and ALV (38), a triplex qPCR for the detection of ALV subgroup J (ALV-J), reticuloendotheliosis virus (REV), and CIAV (39), and a multiplex qPCR for the detection of Marek’s disease virus (MDV), REV, ARV, CIAV, infectious Bursal disease virus (IBDV), and FAdV have been reported (40). However, up to now, no assay has been reported on the quadruplex RT-qPCR for the simultaneous detection of ALV, CIAV, ARV, and FAdV. In this study, the specific primers and probes were designed for the env gene of ALV, the VP1 gene of CIAV, the M1 gene of ARV, and the ORF1 gene of FAdV, and a quadruplex RT-qPCR was successfully established for the differential detection of these four viruses.

## 2 Materials and methods

### 2.1 Reference strains

The vaccine strains were purchased from Harvac Biotechnology Co., LTD (Harbin, China): Newcastle disease virus (NDV), avian influenza virus (AIV, subtype H5, H7, and H9), MDV (strain 814), infectious bronchitis virus (IBV, strain LDT3-A), and IBDV (strain B87).

The positive clinical samples of ALV, CIAV, ARV, FAdV and infectious laryngotracheitis virus (ILTV) were provided by our laboratory.

### 2.2 Clinical samples

From September 2024 to September 2025, a total of 1,575 clinical samples were collected from chickens in 21 breeder farms across 14 cities in Guangxi province, China. Of which, 365 tissue samples (including liver, spleen, and kidney from each chicken, and these tissues from each chicken was taken as one sample when tested using the RT-qPCR) were collected from dead chickens, 560 sera were collected from laying hens, and 650 meconium samples were collected from newly hatched chicks. All samples were stored at −80 °C until use.

### 2.3 Primers and probes

**Figure 1:**
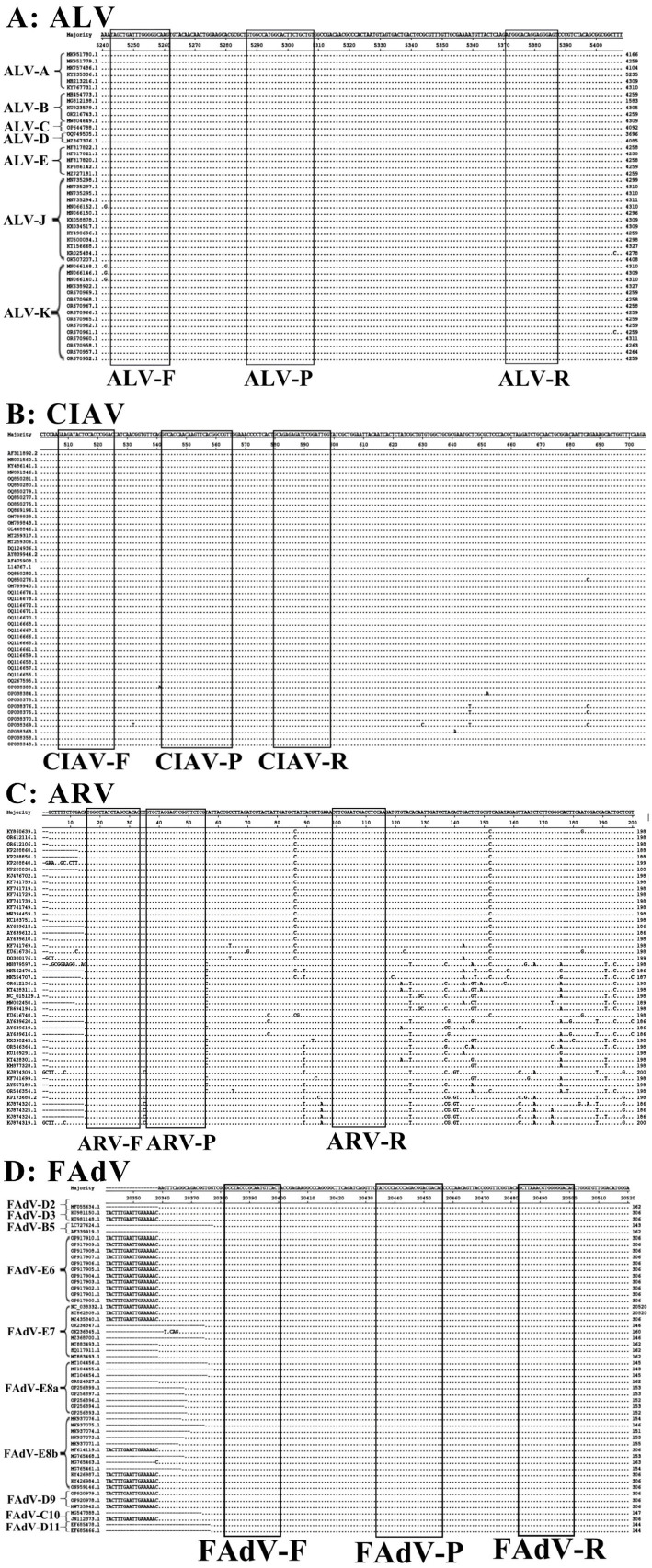
The locations of the designed primers and probes for the quadruplex RT-qPCR. The locations of primers and probes are shown in the nucleotide sequence alignments of the ALV env gene **(A)**, CIAV VP1 gene **(B)**, ARV M1 gene **(C)**, and FAdV ORF1 gene **(D)**, respectively. F, Forward primer; P, TaqMan probe; R, Reverse primer.

The genome sequences of ALV (47 strains), CIAV (47 strains), ARV (47 strains), and FAdV (53 strains) representative strains were downloaded from NCBI GenBank.^1^ These viral strains came from different countries (geographic origins) in different dates, and included different subtypes or genotypes (genetic diversity) of corresponding viruses. The detailed information of these viral strains is shown in Supplementary Tables S1–S4. Multiple sequence alignments were performed (Figure 1), and the primers and probes were designed for amplifying the conserved regions of the ALV env gene, the CIAV VP1 gene, the ARV M1 gene, and the FAdV ORF1 gene (Table 1). This ensured that the developed assay was expected to reliably detect divergent or emerging strains of ALV, CIAV, ARV, and FAdV, and substantially enhanced the confidence of the assay’s broad applicability. The quadruplex RT-qPCR was developed for absolute quantification of viral nucleic acids complying with the MIQE (Minimum Information for Publication of Ouantitative Real-Time PCR Experiments) Guidelines. The development and application of the quadruplex RT-qPCR was performed in Guangxi Center for Animal Disease Control and Prevention, China.

**Table 1:** The designed primers and probes.

Primer/Probe	Sequence (5′ → 3′)	Gene	Tm/°C	Product size (bp)
ALV-F	TAGCTGATTTGGGGGCAAG	env	56.7	145
ALV-R	ACTCCCTCCTGTCCCAT		59.7	
ALV-P	FAM-GTGGCCATGGCACTTCTGCTGT-BHQ1		66.4	
CIAV-F	GAAGATACTCCACCCGGAC	VP1	56.4	92
CIAV-R	CCAATCCGGATCTCTCTGC		56.8	
CIAV-P	ROX-CACCAACAAGTTCACGGCCGTT-BHQ2		64.2	
ARV-F	TGGCCTATCTAGCCACAC	M1	54.5	101
ARV-R	TTGGAGGTCGATTCGAGG		55.6	
ARV-P	CY5-GTGCTAGGAGTCGGTTCTCG-BHQ2		60.0	
FAdV-F	GCCTACCCGCAATGTCACT	ORF1	58.5	120
FAdV-R	CTGTCCCCCACGTTTAAGC		58.7	
FAdV-P	VIC-TATCCCACCCAGACGGACGACAC-BHQ1		60.9	

### 2.4 Extraction of nucleic acids

About 0.3 g (0.1 g of liver, spleen, and kidney each) of tissues were put into a 2.0 mL EP tube. Then, phosphate-buffer saline (PBS, pH7.2, w/v = 1:4) was added, homogenized using Retsch MM400 tissue homogenizer (Haan, Germany), and centrifuged (12,000 rpm, 5 min, 4 °C). About 0.3 g meconium were put into a 2.0 mL EP tube. Then, PBS (pH7.2, w/v = 1:4) was added, vortexed (1 min), and centrifuged (12,000 rpm, 5 min, 4 °C).

Two hundreds of supernatants from tissue or meconium, and 200 μL of serum were used to extract nucleic acids using the TaKaRa MiniBEST Viral RNA/DNA Extraction Kit Ver.5.0 (Code No. 9766, Dalian, China) per the manufacturer’s instructions.

### 2.5 Preparation of the RNA standards

The T7 promoter sequence (TAATACGACTCACTATAGGG) was incorporated at the 5′ end of the forward primer of ALV, ARV (Table 1). The total nucleic acids were extracted from the positive samples of ALV, or ARV using the TaKaRa MiniBEST Viral RNA/DNA Extraction Kit Ver.5.0 (Code No. 9766, Dalian, China), and reverse transcribed into cDNA using the PrimeScript II 1st Strand cDNA Synthesis Kit (Code No. 6210A, TaKaRa, Dalian, China). Using the specific forward/reverse primers, the targeted fragments of the ALV env gene, and ARV M1 gene were amplified using the TaKaRa PrimeScript™ One Step RT-PCR Kit Ver.2.0 (Dye Plus) (Code No. RR057A, Dalian, China) in a 50 μL reaction system (50 °C for 30 min; 94 °C for 2 min; 35 cycles of 94 °C for 30 s, 60 °C for 50 s, and 72 °C for 50 s). The PCR products were used as templates to obtain the RNA standards of ALV, and ARV through *in vitro* transcription using the TaKaRa in vitro Transcription T7 Kit (for siRNA Synthesis) (Code No. 6140, Dalian, China). After the transcription reaction, 10 μL of Recombinant DNase I (RNase-free DNase I) solution (Code No. 2270, TaKaRa, Dalian, China) was added to the reaction mixture (37 °C 30 min). Then, the RNA standards were purified using the SteadyPure RNA Purification Kit (Code No. AG21033, Accurate Biotechnology, Hunan, China). The purified RNA standards were named sr-ALV, and sr-ARV, respectively, and were determined their concentrations using a spectrophotometer (Thermo Fisher, Waltham, MA, USA). The copy number of the RNA standards was calculated using the following formula:


$$\text{Standard}\;(\text{copies}/\mathrm{\mu L})=\frac{\left(6.02\times 10^{23})\times (X\;\mathrm{ng}/\mathrm{\mu L}\times 10^{-9}\right)}{\mathrm{RNA}\;\text{standard length}(\mathrm{bp})\times 340}$$

### 2.6 Construction of the DNA standards

The CIAV, and FAdV nucleic acids were extracted from the positive samples of CIAV, or FAdV using the TaKaRa MiniBEST Viral RNA/DNA Extraction Kit Ver.5.0 (Code No. 9766, Dalian, China). The specific primers of CIAV, and FAdV in Table 1 were used for amplification using DNA as templates. The PCR products were purified using MiniBEST DNA Fragment Purification Kit Ver.4.0 (Code No. 9761, TaKaRa, Dalian, China), cloned into pMD18-T vector (Code No. 6011, TaKaRa, Dalian, China), and transformed into *E. coli* DH5α competent cells (Code No. 9057, TaKaRa, Dalian, China). The positive clones were cultured in LB solution, and the plasmid constructs were extracted using MiniBEST Plasmid Extraction Kit Ver.5.0 (Code No. 9760, TaKaRa, Dalian, China). The DNA plasmid standards were named sd-CIAV, and sd-FAdV, respectively. Their concentrations were determined as abovementioned in Section 2.5. The copy number of the DNA standards was calculated using the following formula:


$$\text{Standard}\;(\text{copies}/\mathrm{\mu L})=\frac{\left(6.02\times 10^{23})\times (X\;\mathrm{ng}/\mathrm{\mu L}\times 10^{-9}\right)}{\text{plasmid length}(\mathrm{bp})\times 660}$$

### 2.7 Optimization of component concentration

A mixture of four RNA/DNA standards (sr-ALV, sd-CIAV, sr-ARV, and sd-FAdV) with a concentration of 10^6^ copies/μL was prepared for primer and probe concentration optimization. The final concentrations of each primer-probe pair were set at 100, 200, 300, 400, and 500 nmol/L, respectively. A matrix approach was employed to systematically combine primers and probes at different concentrations, with three replicates per concentration. The optimal concentration combination was determined according to the RT-qPCR amplification results using the ABI QuantStudio™ 6 real-time system (Carlsbad, California, USA). The combination was selected basing on the lowest Ct value, the highest fluorescence signal (ΔRn), and the optimal amplification curve characteristics.

### 2.8 Optimization of annealing temperature

Based on the optimized RT-qPCR system, the annealing temperature was further optimized. Seven temperature gradients of 57 °C, 58 °C, 59 °C, 60 °C, 61 °C, 62 °C, and 63 °C were set, and three replicates were done for each temperature. The optimal annealing temperature was determined according to the Ct values, the fluorescence signal intensities, and the amplification curve shapes at different temperatures.

### 2.9 Generation of the standard curves

The RNA/DNA standards of sr-ALV, sd-CIAV, sr-ARV, and sd-FAdV were homogenously mixed at a 1:1:1:1 ratio, followed by ten-fold serial dilution ranging from 1.00 × 10^8^ to 1.00 × 10^2^ copies/μL (final reaction concentration), and used as templates to perform quadruplex RT-qPCR. The standard curves were obtained under the optimal reaction conditions.

### 2.10 Analytical specificity

The mixture of four reference RNA/DNA standards of sr-ALV, sd-CIAV, sr-ARV, and sd-FAdV, and the DNA or RNA of AIV-H5, AIV-H7, AIV-H9, NDV, IBV, ITLV, IBDV, and MDV were used as templates to perform quadruplex RT-qPCR using the optimized reaction system and protocol. The specificity was evaluated by observing the amplification curve and Ct value to ensure no false positive result.

### 2.11 Analytical sensitivity

The mixture of four reference RNA/DNA standards of sr-ALV, sd-CIAV, sr-ARV, and sd-FAdV was 10-fold serially diluted ranging from 10^7^ to 10^−1^ copies/μL, and used as templates to analyze the sensitivity of the developed assay. Amplification was carried out using the optimized quadruplex RT-qPCR reaction system and protocol. The limits of detection (LODs) were determined basing on the lowest templates at which amplification curves could be observed in all replicate wells.

In addition, the mixture of four reference RNA/DNA standards sr-ALV, sd-CIAV, sr-ARV, and sd-FAdV with concentration of 500, 250, 125, and 62.5 copies/reaction was used as templates to perform quadruplex RT-qPCR. The LODs were determined through probit regression analysis.

### 2.12 Repeatability analysis

To evaluate the repeatability (within-batch precision) and reproducibility (between-batch precision) of the quadruplex RT-qPCR, the reference RNA/DNA standards of three different concentrations (10^6^, 10^4^, and 10^2^ copies/μL) were used to evaluate within-batch and between-batch variabilities. For within-batch variability, each dilution was repeated three times on the same experiment. For between-batch variability, three independent experiments were conducted in different days. The coefficient of variation (CVs) of the Ct values were calculated.

### 2.13 Evaluation of the clinical samples

To evaluate the application feasibility of the established quadruplex RT-qPCR, it was used to test the 1,575 clinical samples (tissue, serum, and meconium), which were collected from 14 cities (Nanning, Liuzhou, Chongzuo, Qinzhou, Baise, Yulin, Beihai, Hechi, Guigang, Qinzhou, Laibin, Hezhou, Fangchenggang, and Guilin) in Guangxi province, southern China during 2024–2025.

In addition, these samples were also tested for ALV, CIAV, ARV, and FAdV using RT-qPCR method in the following reference methods: Diagnostic techniques for avian leukosis (National Standard of the People's Republic of China),^2^ Diagnostic techniques for chicken infectious anemia (Agricultural Industry Standard of the People's Republic of China),^3^ Diagnostic techniques for avian viral arthritis (Agricultural Industry Standard of the People's Republic of China),^4^ and Diagnostic methods for group I fowl adeonvirus (Agricultural Industry Standard of the People's Republic of China).^5^ The detection results of the developed assay and the reference assays were compared, and the clinical sensitivity and specificity was evaluated.

### 2.14 Statistical methods

The data of LOD, clinical sensitivity, clinical specificity, coincidence rate (agreement), and hit rate were calculated and determined as follows.

The LOD determined using Probit regression analysis is a statistical estimate based on a probability model. It takes response variability into account and explicitly links concentration with detection probability. It is more rigorous than simple signal-to-noise ratio methods, particularly suitable for situations where the response-concentration relationship is nonlinear. It serves as a robust tool in analytical chemistry and bioassays for determining method sensitivity. The probit regression calculation formula is as follows (a = intercept term, b = slope):


$$\text{Probit}\;(p)=a+b\times \log_{10}(\mathrm{LOD})$$

To compare the established assay and the reference reported assays, the clinical sensitivity and specificity of the established assay were determined. The clinical sensitivity is a core indicator for comparing the test performance of two detection methods. When comparing the sensitivity of two methods (A vs. B), the key is to determine whether the observed difference is real and reliable, rather than due to random error. In this case, it is not sufficient to compare only point estimates; their 95% confidence intervals must also be compared. The clinical sensitivity (95% CI) calculation formula is as follows (TP = True positive, FP = false positive, FN = false negative):


$$\text{Clinical sensitivity}\;(95\%\mathrm{CI})=\frac{\mathrm{TP}}{\mathrm{TP}+\mathrm{FN}}\pm 1.96\times \sqrt{\frac{\mathrm{TP}\times \mathrm{FN}}{{\left(\mathrm{TP}+\mathrm{FN}\right)}^{3}}}$$

A test with high specificity is an excellent “confirmatory” tool. When comparing two detection methods, it is essential to examine the 95% confidence interval of the difference in their specificities: if the interval is entirely greater than 0, one method is significantly superior; if it includes 0, there is no statistically significant difference. Specificity and sensitivity often involve a trade-off; only by combining both, along with their confidence intervals, can the overall test performance be comprehensively evaluated. The clinical specificity (95% CI) calculation formula is as follows (TN = True negative, FP = false positive, FN = false negative):


$$\text{Clinical specificity}\;(95\%\mathrm{CI})=\frac{\mathrm{TN}}{\mathrm{TN}+\mathrm{FP}}\pm 1.96\times \sqrt{\frac{\mathrm{TN}\times \mathrm{FP}}{{\left(\mathrm{TN}+\mathrm{FP}\right)}^{3}}}$$

Consistency measures the degree of agreement between two measurements obtained by the same detection method (or by different observers or different methods). It is commonly quantified using percentage agreement or the Kappa statistic. The agreement calculation formula is as follows (TP = True positive, TN = True negative, N = total):


$$\text{Agreement}\;(\%)=\frac{\mathrm{TP}+\mathrm{TN}}{N}$$

Hit rate is suitable for quickly understanding overall performance. However, it must be analyzed in conjunction with sensitivity and specificity to comprehensively and fairly evaluate diagnostic performance. The hit rate calculation formula is as follows (TP = True positive, TN = True negative, FP = false positive, FN = false negative):


$$\mathrm{Hit}\;\text{rate}=\frac{\mathrm{TP}+\mathrm{TN}}{\mathrm{TP}+\mathrm{TN}+\mathrm{FP}+\mathrm{FN}}$$

## 3 Results

### 3.1 Construction of the RNA standards

The total RNA was extracted from the ALV, and ARV positive clinical samples, respectively, and taken as templates for PCR amplification of the env gene of ALV, and the M1 gene of ARV. The amplified fragments were subjected to *in vitro* transcription to obtain large amounts of RNA. The synthetic RNAs were purified and named sr-ALV, and sr-ARV, respectively. They were used as the RNA standards for development of the quadruplex RT-qPCR. Their original concentrations were determined to be 1.45 × 10^10^, and 1.73 × 10^10^ copies/μL, respectively, then were diluted to 1.00 × 10^9^ copies/μL and stored at −80 °C until use.

### 3.2 Construction of the DNA standards

The total DNA were extracted from the CIAV, and FAdV positive samples, respectively. After PCR amplification of the VP1 gene of CIAV, and the ORF1 gene of FAdV, the target fragments were purified to construct the plasmid DNA standard. The positive clones were cultured, and the recombinant plasmid constructs were extracted and named sd-CIAV, and sd-FAdV, respectively. Their initial concentrations were determined to be 1.04 × 10^10^, and 1.29 × 10^10^ copies/μL, respectively, then were diluted to 1.0 × 10^9^ copies/μL and stored at −80 °C until use.

### 3.3 Attainment of the reaction conditions

Through iterative optimization of the experimental conditions, the optimal parameters for the quadruplex RT-qPCR assay were determined, including the primer and probe concentrations, annealing temperature, and reaction cycles. The total reaction volume of 20 μL with different components and optimal parameters are shown in Table 2. The reaction protocol comprised the following steps: 42 °C for 5 min, 95 °C for 10 s; followed by 40 cycles of 5 s at 95 °C and 30 s at 57 °C, with fluorescence signal acquisition at the end of each cycle. The samples with Ct values ≤ 36 were determined as positive samples.

**Table 2:** The components and the optimal parameters.

Ingredient	Volume (μL)	Final concentration (nM)
2 × One-Step RT-PCR Buffer III	10	/
Ex Taq HS (5 U/μL)	0.4	/
PrimerScript RT Enzyme Mix	0.4	/
ALV-F (20 pmol/μL)	0.3	300
ALV-R (20 pmol/μL)	0.3	300
ALV-P (20 pmol/μL)	0.3	300
CIAV-F (20 pmol/μL)	0.5	500
CIAV-R (20 pmol/μL)	0.5	500
CIAV-P (20 pmol/μL)	0.5	500
ARV-F (20 pmol/μL)	0.5	500
ARV-R (20 pmol/μL)	0.5	500
ARV-P (20 pmol/μL)	0.5	500
FAdV-F (20 pmol/μL)	0.3	300
FAdV-R (20 pmol/μL)	0.3	300
FAdV-P (20 pmol/μL)	0.3	300
Nucleic acid template	2.0	/
Nuclease-free distilled water	2.4 (Up to 20)	/

### 3.4 Generation of the standard curves

**Figure 2:**
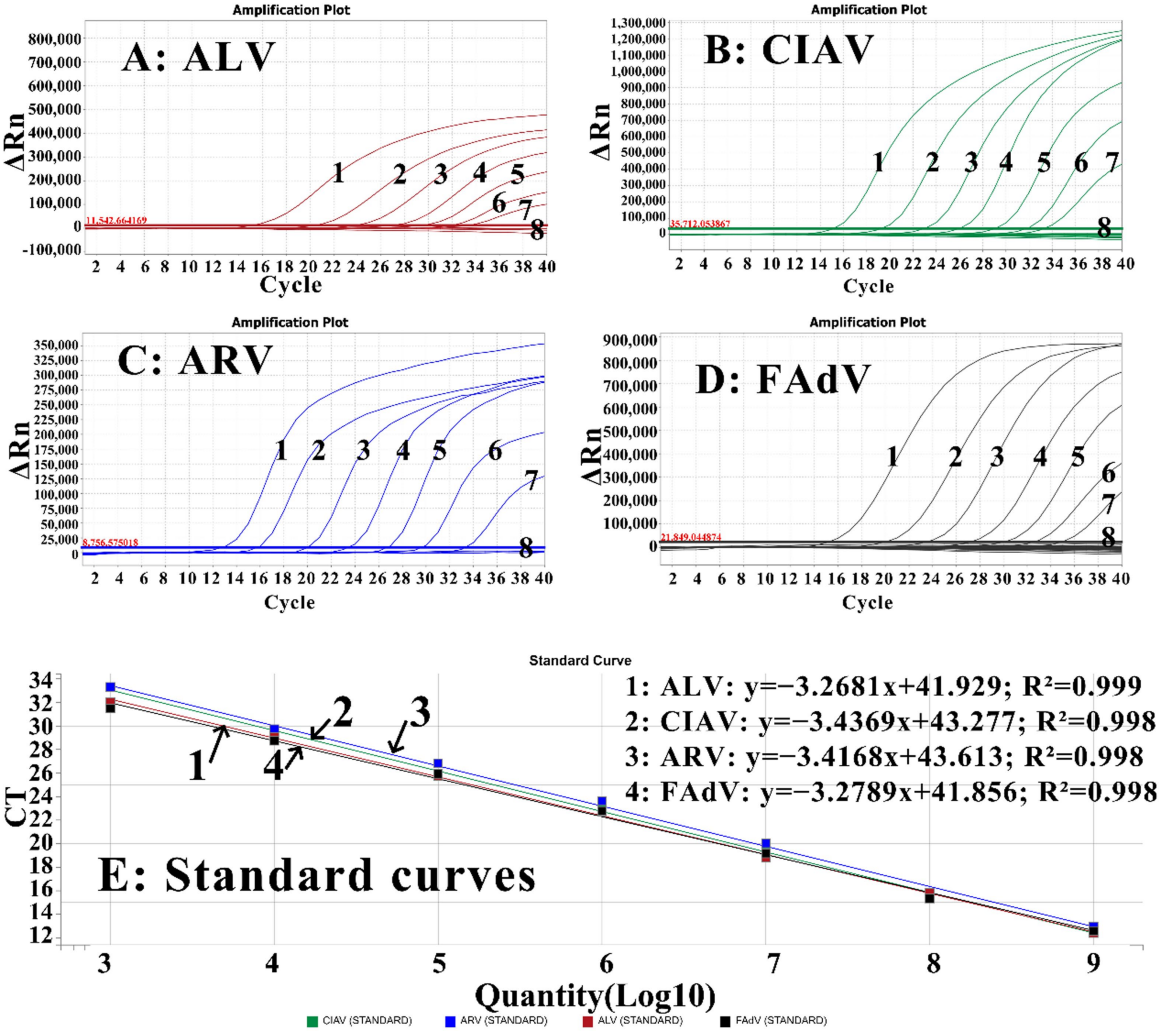
The amplification curves of ALV **(A)**, CIAV **(B)**, ARV **(C)**, and FAdV **(D)**, and the standard curves **(E)** of the quadruplex RT-qPCR. In **(A–D)**, 1–7: the concentrations of the RNA/DNA standards ranging from 10^8^ to 10^2^ copies/μL were used to generate the standard curves; 8: nuclease-free distilled water as negative control. In **(E)**, 1–4: the generated standard curves of ALV, CIAV, ARV, and FAdV, respectively.

Four reference RNA/DNA standards (sr-ALV, sd-CIAV, sr-ARV, and sd-FAdV) were mixed at ratio of 1:1:1:1, and 10-fold serially diluted from 1.00 × 10^9^ to 1.00 × 10^3^ copies/μL (the final reaction concentrations ranging from 1.00 × 10^8^ to 1.00 × 10^2^ copies/μL). They were used as templates to perform quadruplex RT-qPCR for obtaining the corresponding standard curves. The results indicated that ALV (slope = −3.2681, R^2^ = 0.999, Eff% = 102.301), CIAV (slope = −3.4369, R^2^ = 0.998, Eff% = 97.115), ARV (slope = −3.4168, R^2^ = 0.998, Eff% = 99.145), and FAdV (slope = −3.2789, R^2^ = 0.998, Eff% = 103.523) demonstrated excellent correlation coefficients (R^2^ ≥ 0.998) and amplification efficiencies (Eff% ≥ 97.115) (Figure 2).

### 3.5 Specificity

**Figure 3:**
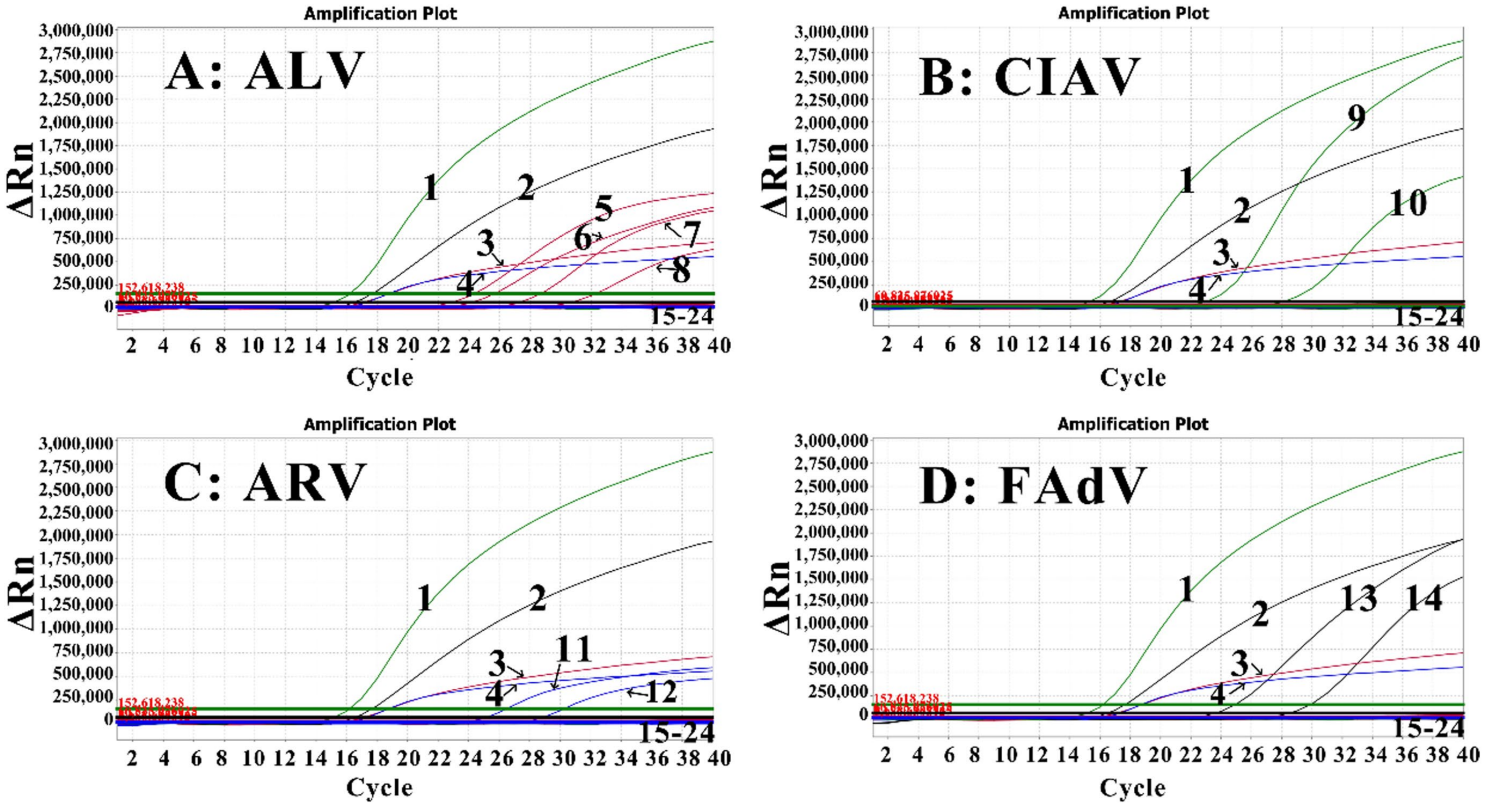
Specificity assessment of the quadruplex RT-qPCR for ALV **(A)**, CIAV **(B)**, ARV **(C)**, and FAdV **(D)**. 1: CIAV; 2: FAdV; 3: ALV; 4: ARV; 5–8: The positive clinical samples of subtype A, B, J, and K of ALV; 9, 10: the positive clinical samples of CIAV; 11, 12: the positive clinical samples of ARV; 13, 14: the clinical samples of serotype E8a and E8b of FAdV; 15–22: AIV-H5, AIV-H7, AIV-H9, NDV, IBV, ITLV, IBDV, and MDV, respectively; 23: the negative sample; 24: distilled water as negative control.

The four RNA/DNA standards (sr-ALV, sd-CIAV, sr-ARV, and sd-FAdV), and the RNA or DNA of AIV-H5, AIV-H7, AIV-H9, NDV, IBV, IBDV, MDV, and ILTV were used as templates to evaluate the specificity of the developed assay. The results demonstrated that the established assay generated specific amplification curves corresponding to ALV, CIAV, ARV, and FAdV, without amplification curve for other common chicken viruses, indicating excellent specificity (Figure 3).

### 3.6 Sensitivity

**Figure 4:**
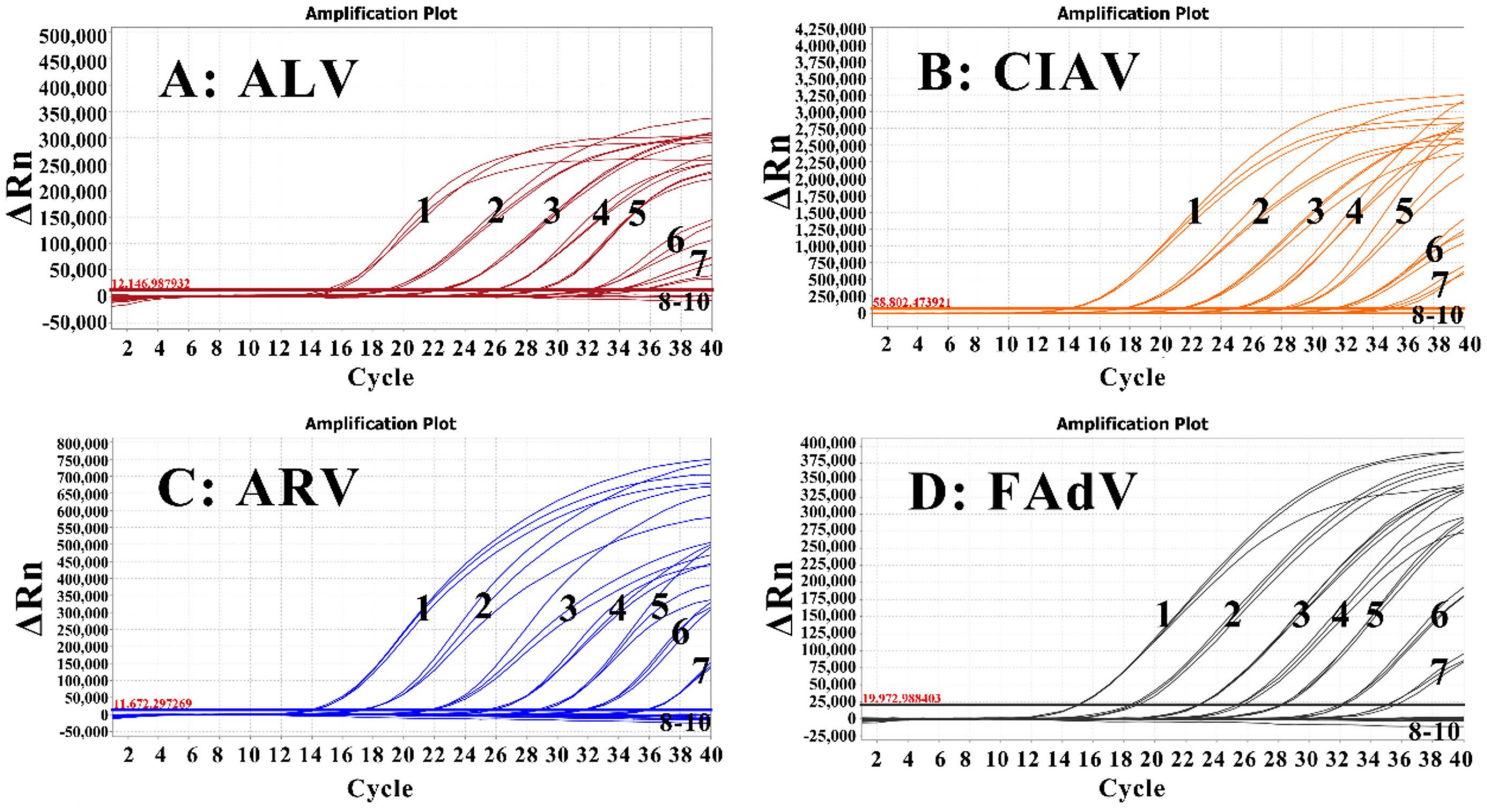
Sensitivity assessment of the quadruplex RT-qPCR for ALV **(A)**, CIAV **(B)**, ARV **(C)**, and FAdV **(D)**. In **(A–D)**, 1–9: The RNA/DNA standards with final reaction concentrations ranging from 10^7^ to 10^−1^ copies/μL were used to analyze the sensitivity of the quadruplex RT-qPCR; 10: Negative control.

The four RNA/DNA standards were mixed at ratio of 1:1:1:1, 10-fold serially diluted from 1.00 × 10^7^ to 1.00 × 10^−1^ copies/μL (final reaction concentration), and used as templates for the quadruplex RT-qPCR. The results indicated that the LODs were determined to be 10^1^ copies/μL for ALV, CIAV, ARV, and FAdV (Figure 4).

**Figure 5:**
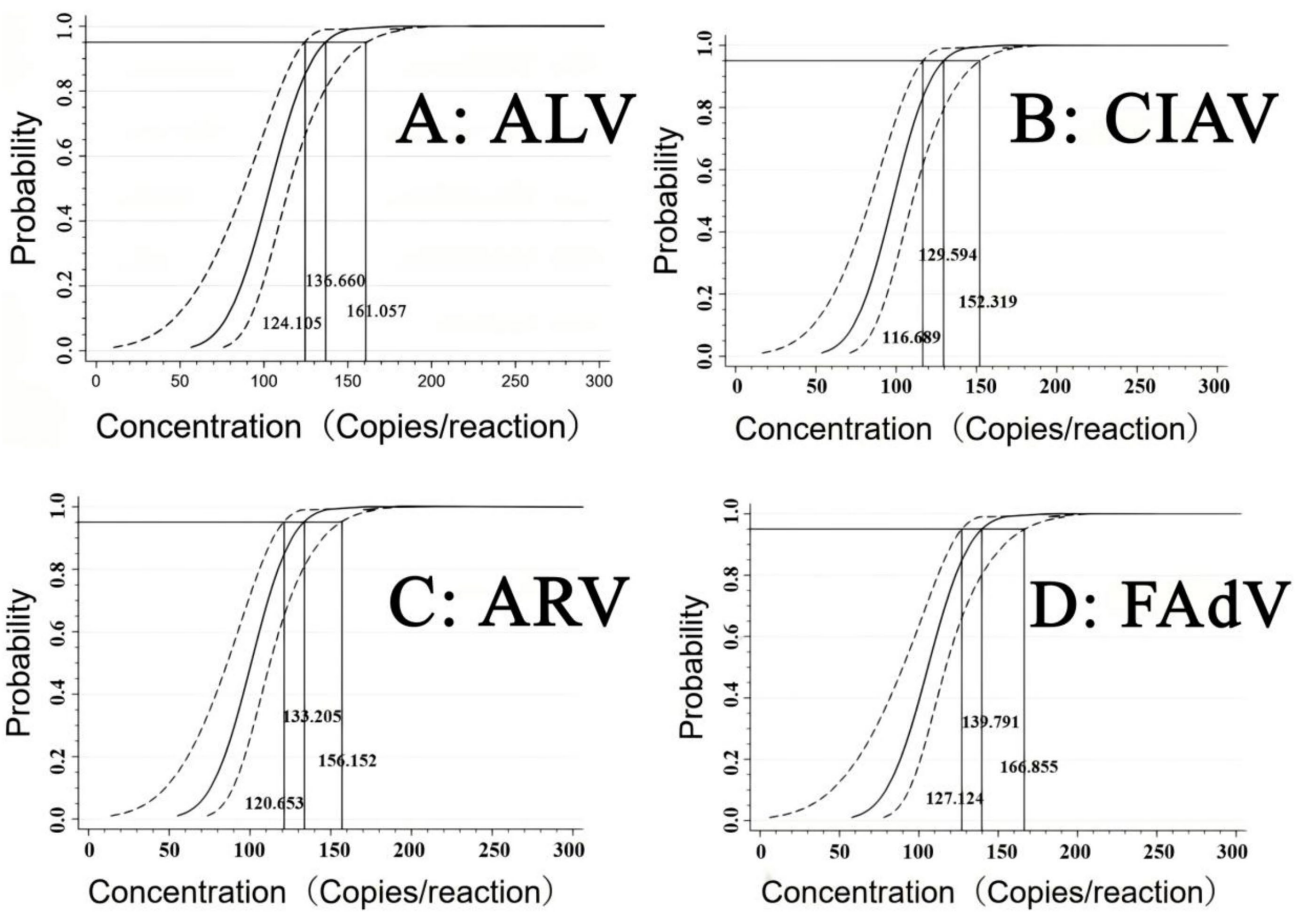
Sensitivity assessment using probit regression analysis. The quadruplex RT-qPCR showed high sensitivity for ALV **(A)**, CIAV **(B)**, ARV **(C)**, and FAdV **(D)** with 136.66, 129.59, 133.21, and 139.79 copies/reaction, respectively.

Furthermore, the four RNA/DNA standard mixture was diluted to 500, 250, 125, and 62.5 copies/reaction, and used as templates for the quadruplex RT-qPCR. The software IBM SPSS (Armonk, NY, USA, accessed on 27 October 2024) was used to analyze the probability regression and determine the LODs of ALV, CIAV, ARV, and FAdV. The results demonstrated that the LODs of ALV, CIAV, ARV, and FAdV were 136.66 (95% confidence interval (CI) of 124.11–161.06), 129.59 (95% CI of 116.69–152.32), 133.21 (95% CI of 120.65–156.15), and 139.79 (95% CI of 127.12–166.86) copies/reaction, respectively (Table 3; Figure 5).

**Table 3:** The Ct values and hit rates of the RNA/DNA standards with serial dilution.

RNA/DNA standard	Copies/reaction	Number of samples	Quadruplex RT-qPCR	
			Ct value (Average)	Hit rate (%)
sr-ALV	500	24	34.14	100
	250	24	35.36	100
	125	24	35.92	87.50
	62.5	24	ND	0
sd-CIAV	500	24	34.19	100
	250	24	34.77	100
	125	24	35.90	95.83
	62.5	24	ND	0
sr-ARV	500	24	34.01	100
	250	24	35.18	100
	125	24	35.96	91.67
	62.5	24	ND	0
sd-FAdV	500	24	34.07	100
	250	24	34.99	100
	125	24	35.90	83.33
	62.5	24	ND	0

### 3.7 Repeatability

The four RNA/DNA standards of three concentrations (1.0 × 10^6^, 1.0 × 10^4^, and 1.0 × 10^2^ copies/μL) were used as templates to evaluate the repeatability of the quadruplex RT-qPCR assay. The results demonstrated that the intra-batch and inter-batch CVs were 0.29–0.93% and 0.29–0.99%, respectively, indicating excellent repeatability of the developed assay (Table 4).

**Table 4:** Repeatability of the quadruplex RT-qPCR.

RNA/DNA standard	Concentration (Copies/μL)	Ct value of intra-assay			Ct value of inter-assay		
		x̄	SD	CV (%)	x̄	SD	CV (%)
sr-ALV	1.0 × 10^6^	18.23	0.18	0.93	18.13	0.18	0.94
	1.0 × 10^4^	25.74	0.21	0.80	25.74	0.21	0.80
	1.0 × 10^2^	31.88	0.25	0.78	31.87	0.25	0.78
sd-CIAV	1.0 × 10^6^	18.84	0.43	0.30	18.82	0.08	0.43
	1.0 × 10^4^	25.74	0.08	0.30	25.43	0.08	0.32
	1.0 × 10^2^	33.04	0.13	0.38	32.88	0.12	0.38
sr-ARV	1.0 × 10^6^	19.83	0.08	0.41	19.67	0.19	0.96
	1.0 × 10^4^	26.48	0.17	0.64	26.43	0.08	0.31
	1.0 × 10^2^	33.03	0.16	0.49	32.87	0.13	0.38
sd-FAdV	1.0 × 10^6^	18.93	0.05	0.32	19.48	0.20	0.99
	1.0 × 10^4^	26.03	0.08	0.31	26.03	0.09	0.36
	1.0 × 10^2^	31.98	0.09	0.29	31.98	0.10	0.29

### 3.8 Assessment of the clinical samples

The 1,575 clinical samples of tissues, sera and meconium from 21 breeder farms in 14 cities of Guangxi province were tested using the established quadruplex RT-qPCR. The results demonstrated that the positivity rates of ALV, CIAV, ARV, and FAdV were 36.89% (581/1,575), 17.65% (278/1,575), 2.16% (34/1,575) and 7.05% (111/1,575), respectively. The co-infection of these pathogens was existed in these samples, of which the highest double co-infection rate of ALV and CIAV was 15.49% (244/1,575), the highest rate of triple infection was ALV + CIAV + FAdV with 4.06% (64/1,575), and the rate of quadruple co-infection of ALV + CIAV + ARV + FAdV was 0.57% (9/1,575) (Table 5).

**Table 5:** The test results of the clinical samples from 14 cities in Guangxi province, China.

Region	Number	Number of positive samples													
		ALV	CIAV	ARV	FAdV	ALV+CIAV	ALV+ARV	ALV+FAdV	CIAV+ARV	CIAV+ FAdV	ARV+ FAdV	ALV+CIAV+ FAdV	ALV+ARV+ FAdV	CIAV+ARV+ FAdV	ALV+CIAV+ARV+ FAdV
Nanning	80	34	11	2	2	11	2	2	1	1	1	1	0	0	0
Liuzhou	30	17	8	1	6	7	1	5	1	3	1	3	1	1	1
Guilin	224	45	29	8	4	19	7	4	7	4	2	3	2	2	2
Wuzhou	92	38	18	1	12	16	1	12	1	10	1	9	1	1	1
Beihai	92	35	26	0	19	20	0	15	0	8	0	7	0	0	0
Chongzuo	30	13	10	4	1	10	4	1	0	1	1	1	0	0	0
Laibin	96	36	25	0	18	24	0	17	0	10	0	8	0	0	0
Hezhou	111	30	10	1	5	10	1	5	1	5	1	5	1	1	1
Yulin	340	135	50	12	9	44	11	9	9	4	3	3	2	2	1
Baise	53	18	13	0	8	13	0	8	0	7	0	7	0	0	0
Hechi	142	61	35	0	8	30	0	7	0	4	0	4	0	0	0
Qinzhou	215	95	30	4	11	28	4	11	4	8	3	8	3	2	2
Fangchenggang	30	12	11	0	7	10	0	7	0	5	0	4	0	0	0
Guigang	40	12	2	1	1	2	1	1	1	1	1	1	1	1	1
Total	1,575	581	278	34	111	244	32	104	25	71	14	64	11	10	9
Positivity rate (%)		36.89	17.65	2.16	7.05	15.49	2.03	6.6	1.59	4.51	0.89	4.06	0.7	0.63	0.57

In addition, the 1,575 clinical samples were tested for ALV, CIAV, ARV, and FAdV using the reported reference methods. The positivity rates of ALV, CIAV, ARV, and FAdV were 36.83% (580/1,575), 17.59% (277/1,575), 2.16% (34/1,575), and 6.98% (110/1,575), respectively. Compared with the reference methods, the clinical sensitivity and specificity of the developed assay for ALV, CIAV, ARV, and FAdV were 99.05 and 99.45%, 99.46 and 99.88%, 100.00 and 100.00%, 100.00 and 99.97%, respectively. The coincidence rates of these methods were all higher than 99.31% (Table 6).

**Table 6:** The clinical sensitivity and specificity of the established assay.

The established assay		The reference assay		Total	Clinical sensitivity (95% CI)	Clinical specificity (95% CI)	Agreement
		Positive	Negative				
ALV	Positive	575	6	581	99.05% (98.26–99.84%)	99.45% (98.73–99.76%)	99.31%
	Negative	5	989	994			
	Total	580	995	1,575			
CIAV	Positive	276	2	278	99.46% (98.12–99.82%)	99.88% (99.65–99.93%)	99.81%
	Negative	1	1,296	1,297			
	Total	277	1,298	1,575			
ARV	Positive	34	0	34	100% (91.57–100.00%)	100% (99.74–100.00%)	100.00%
	Negative	0	1,541	1,541			
	Total	34	1,541	1,575			
FAdV	Positive	110	1	111	100% (96.70–100.00%)	99.97% (99.72–99.98%)	99.94%
	Negative	0	1,464	1,464			
	Total	110	1,465	1,575			

Since the clinical samples included tissue, serum, and meconium, it was further analyzed to evaluate the influence of different sample types on the positivity rates of these four viruses (Table 7). The results indicated that the positivity rates of ALV, CIAV, ARV, and FAdV in different sample types were similar, without significant difference (*p* > 0.05).

**Table 7:** The positivity rates of ALV, CIAV, ARV, and FAdV in different sample types.

Sample type	Positive samples			
	ALV (%)	CIAV (%)	ARV (%)	FAdV (%)
Tissue	137/365 (37.53%)	69/365 (18.90%)	10/365 (2.74%)	29/365 (7.95%)
Serum	201/560 (35.89%)	95/560 (16.96%)	10/560 (1.79%)	36/560 (6.43%)
Meconium	243/650 (37.38%)	114/650 (17.54%)	14/650 (2.50%)	46/650 (7.07%)
Total	581/1,575 (36.89%)	278/1,575 (17.65%)	34/1,575 (2.16%)	111/1,575 (7.05%)

## 4 Discussion

ALV, CIAV, ARV, and FAdV are important pathogens that can spread through horizontal and vertical transmission, and are prevalent in many countries around the world. They induce immunosuppression (2, 41–43), and aggravate the clinical signs and damages of the diseases, especially while co-infection with other pathogens (26–29). These viruses have posed huge economic pressure to the poultry industry worldwide. Chickens infected with ALV, CIAV, ARV, and FAdV usually exhibit similar clinical symptoms, such as diarrhea, jaundice, and anemia (1, 2, 8, 13, 20). Since these signs lack specificity, it is difficult to make an accurate differential diagnosis based solely on clinical symptoms. What is more, these viruses usually co-infect with other pathogens in the clinical cases, making the clinical symptoms more complex and changeable, which further increasing the difficulty of differential diagnosis (26–29, 44). To accurately identify and distinguish these viruses, some technical methods have been developed, including virus isolation and identification, electron microscopy observation, serological detection, and nucleic acid testing, etc. Among them, the nucleic acid-based detection methods, such as RT-qPCR technology, have become one of the commonly used detection methods in laboratories due to their high sensitivity and specificity (30, 31). The continuous development and optimization of these laboratory techniques have provided strong support for the early diagnosis, prevention and control of avian viral diseases.

Single RT-qPCR methods for ALV, CIAV, ARV, or FAdV have been reported (33–37). A triplex RT-qPCR for FAdV type I, III and ALV (38), a triplex qPCR for ALV-J, REV, and CIAV (39), and a multiplex RT-qPCR for six viruses of ARV, CIAV, FAdV, MDV, REV and IBV (40) have also been reported. The abovementioned singleplex assays could specifically detect the four viruses with sensitivity for 5 × 10^5^ copies/μL of ALV (33), 82 × 10^2^ copies/μL of CIAV (34), 10 copies/μL of ARV (35), and 6.73 × 10^2^ copies/reaction of FAdV (36), respectively. In addition, the triplex qPCR showed sensitivity of 0.1 TCID_50_/μL for FAdV type I/type III/ALV (38), and 10 copies/μL for ALV-J/REV/CIAV (39). Recently, Karabağ et al. (37) reported the successful diagnosis of ALV-J in asymptomatic commercial layer flocks using qPCR, highlighting the importance of sensitive molecular tools for subclinical detection and surveillance. Especially, co-infection with different pathogens, such as ALV, CIAV, ARV, and FAdV etc., are still common in poultry flocks in China. The multiplex RT-qPCR is a specific, sensitive, and reliable method that is suitable for detection of these pathogens, especially for the early stage of infection with low viral loads, the co-infected cases, and the poultry flocks with subclinical or asymptomatic infections.

However, there have no reports on quadruplex RT-qPCR for the simultaneous detection of ALV, CIAV, ARV, and FAdV to date. In this study, to establish a quadruplex RT-qPCR, multiple sequence alignment analyses were performed using the genome sequences of representative strains of these four viruses from different countries, and the conserved regions in the genomes were selected as the targeted regions for amplification. This strategy helps to enhance the universality of the detection method, enabling it to detect multiple variant strains. The RNA and DNA standards were constructed, used as the positive controls, and showed similar amplification efficiency in the development and validation of the quadruplex RT-qPCR. The systematic optimization of the reaction conditions, including concentrations of primers and probes, annealing temperature and reaction cycles, were performed and determined through a series of gradient experiments and repeated validations. This optimization process not only enhanced the sensitivity of the detection method but also effectively reduced the risk of non-specific amplification. After optimizing the reaction parameters, the quadruplex RT-qPCR for the detection of ALV, CIAV, ARV, and FAdV was successfully established. It indicated high sensitivity with LODs of about 130 copies/reaction, excellent repeatability with intra- and inter-assay CVs of less than 0.99%, and strong specificity without cross-reaction with other control avian viruses. The application of the developed assay was confirmed via testing 1,575 clinical samples, and it showed agreement rates higher than 99.31% of the detection results with those of the reference methods. These results demonstrated that the developed assay was highly specific and sensitive, easy to operate, and obtained the detection results within a short time. To our best knowledge, this is the first report on the multiplex RT-qPCR for the simultaneous detection of ALV, CIAV, ARV, and FAdV. It filled the technological gap in the field of high-throughput and comprehensive molecular diagnostic tools, and met the urgent needs of the breeding industry and veterinary laboratories for efficient pathogen screening tools. Opposed to the single or serial PCR for detection, the quadruplex RT-qPCR in this study integrated four independent assays into one unified workflow, achieving the purpose of sensitive and specific detection of four pathogens in one reaction within 2–3 h. Compared with the singleplex PCR, this assay obtained an approximate 75% reduction in reagents, manual labor, and sample volume, which was suitable for high-throughput detection and large-scale epidemiological surveillance in practices. Compared with the conventional serological techniques (e.g., ELISA), this assay can detect the viral nucleic acids in the early stage of infection, differentiate the vaccine and wild-type strains through specific primers and probes, and attain the capacity of viral load quantification—a critical feature that serological assays cannot provide.

The 1,575 clinical samples from Guangxi province, China were tested for ALV, CIAV, ARV, and FAdV using the developed quadruplex RT-qPCR. The positivity rates of ALV, CIAV, ARV, and FAdV were 36.89, 17.65, 2.16, and 7.05%, respectively, and there existed co-infection of these viruses (Table 5). Interestingly, the positivity rates of ALV, CIAV, ARV, and FAdV from tissue, serum, and meconium were similar for the same virus (Table 7), which indicated that the positivity rates in different types of samples might be different in certain collected times and certain poultry farms. However, the positivity rates in different types of samples would be similar, given the large sample size, indicating that the detection performance might be similar among sample types, and tissue, serum, and meconium could be used for clinical surveillance of ALV, CIAV, ARV, and FAdV, without sample-type-specific performance. The results indicated that these viruses are commonly prevalent in poultry flocks in Guangxi province. Furthermore, they have also been reported in different provinces in China, indicating the high prevalence of these viruses in poultry flocks.

Reports have shown that ALV has high positivity rate in poultry flocks in different provinces in China. In some areas of China, the positivity rates of ALV in the large-scale poultry farms reach from 13.03% up to 72.44% (2). The free-range farmers have a higher risk of infection due to poorer biosecurity conditions, and might have higher incidence rates. The areas with high concentrations of poultry farming are the high-risk areas for the epidemic of ALV due to the high density of poultry and the frequent circulation of live poultry (3). The survey in 11 provinces in China confirmed that ALV-J was the main subtype with detection rates ranging from 3.14% in Guizhou province to 36.96% in Guangxi province, which mainly caused hemangioma and myeloid tumors in broilers (2). In addition, ALV-A and ALV-B are more commonly found in laying hens, while ALV-E is widely present in commercial chicken flocks (4). It is noteworthy that the recombinant subtypes (such as J/A recombinant) have been detected (45), which has increased the genetic diversity of the virus and the difficulty of prevention and control of the disease.

CIAV includes four main groups (A, B, C and D), and Group C is further subdivided into three subgroups (C1-C3). The 350 samples collected from 8 broiler farms in Guangxi province during 2018–2020 showed that subgroup C3 is the predominant genotype with a positivity rate of 17.1% (46). The 1,187 chicken flock samples collected in China during 2017–2019 showed 15.1% (179/1,187) infection rate of CIAV (40). CIAV cause severe immunosuppression to the infected chickens, and the co-infection with other viruses such as ALV usually enhances the immunosuppressive effect and synergistically aggravate the pathogenicity of various viruses (47). In China, there still no commercial vaccine for CIAV, so its prevention and control mainly rely on improving feeding management and hygiene measures.

ARV is widely distributed throughout the world. The 2,340 samples collected from 16 provinces in China from 2019 to 2020 showed the positivity rate of ARV with 4.83% (112/2,340) (48). During the evolution of ARV, the interaction of multiple factors has promoted the formation of new genotypes (19). Among them, the rapid accumulation of point mutations, antigenic drift caused by gene recombination, and the widespread use of vaccines are the main driving forces for the emergence of new genotypes of ARV, posing a huge challenge to the prevention and control of ARV.

FAdV mainly affects broilers aged 3 to 6 weeks, with an extremely high mortality rate, which can reach up to 80%. FAdV is classified into 12 serotypes, among which FAdV-8a, FAdV-8b and FAdV-11 can cause IBH (20–22). During 2007–2018, the most prevalent serotypes of FAdV in China were FAdV-8a and FAdV-8b. The 1,920 clinical samples from 8 provinces in eastern China indicated that chickens had the infection rate of FAdV with 13.28% (255/1,920) (49). Although FAdV vaccines have been widely used, the disease is still highly prevalent in various regions of China (50).

The high prevalence and co-infection rate of ALV, CIAV, ARV, and FAdV pose a serious threat to the production safety of the poultry industry. It is vital to rapid and precisely detect and differentiate these viruses for accurate diagnosis of these diseases, which helps to timely and effectively prevent and control them. The developed quadruplex RT-qPCR in this study is a useful method for the rapid and accurate detection and differentiation of ALV, CIAV, ARV, and FAdV. Especially, according to the detection results of the clinical samples by the developed quadruplex RT-qPCR, the poultry farms can determine whether to use vaccine or not for prevention and control. Furthermore, the poultry farms can select the positive samples for sequence analysis and determine the specific serotypes and genotypes of the prevalent viral strains in order to determine and use the effective vaccine strains.

It is noteworthy that the clinically prevalent ALV, CIAV, ARV, and FAdV strains, especially ALV and ARV as RNA viruses, showing high genetic diversity with different subtypes (genotypes) of epidemic strains. In addition, these viruses are constantly mutating due to persistent immune stress. These might lead to primer mismatches and missed detection of clinically positive samples. These situations require continuous clinical surveillance to obtain the genetic characteristics of the current epidemic strains, in order to ensure the designed primers and probes are specific, accurate, and reliable for the prevalent viral strains.

## 5 Conclusion

A quadruplex RT-qPCR assay was successfully developed in this study, which provided a rapid, sensitive and efficient method for the differential detection of ALV, CIAV, ARV, and FAdV. This method could accurately detect these viruses in the clinical samples. The detection results of the clinical samples indicated that ALV, CIAV, ARV, and FAdV were still commonly prevalent in Guangxi province, China.

## Data Availability

The raw data supporting the conclusions of this article are included in the article.

## Footnotes

1 https://www.ncbi.nlm.nih.gov/nucleotide/, accessed on September 17, 2024.

2 https://openstd.samr.gov.cn/bzgk/std/newGbInfo?hcno=CDF77453514D036215BD799342367470, accessed on September 17, 2024.

3 https://std.samr.gov.cn/hb/search/stdHBDetailed?id=A7B31164793F3AA4E05397BE0A0A2AA6, accessed on September 17, 2024.

4 https://std.samr.gov.cn/db/search/stdDBDetailed?id=2B2A1319575BB26FE06397BE0A0AE4E2, accessed on September 17, 2024.

5 https://hbba.sacinfo.org.cn/stdDetail/0c9ed07cbe3b38f0129c0e420923580f735e9a77385672194f3339821d35b8fb, accessed on September 17, 2024.
