# Supplementary material for: Development of a quadruplex RT-qPCR for the detection of avian leukosis virus, chicken infectious anemia virus, avian reovirus, and fowl adenovirus
Source: Front Vet Sci. 2026 Mar 18;13:1747413. doi: 10.3389/fvets.2026.1747413 (PMC13040548; doi:10.3389/fvets.2026.1747413)
Supplement: Supplementary file 1 [file Data_Sheet_1.pdf]

Supplementary Materials

TABLE S1 The ALV reference strains used in this study

Strain	Area	Date	Accession No.	Subtype
HB18XH01	China	2018	MN735298.1	J
GX16YL01	China	2016	MN735297.1	J
GX16NN03	China	2016	MN735295.1	J
GX15JL01	China	2015	MN735294.1	J
GD15MM01	China	2019	MN066152.1	J
GX15MM61	China	2019	MN066150.1	J
GX16YL01	China	2019	MN066148.1	K
GX16ZS01	China	2019	MN066146.1	K
GX18NN02	China	2019	MN066140.1	K
S0322C	China	2019	MK951780.1	A
S0322B	China	2018	MK951779.1	A
GifN_001	Japan	2015	MK757486.1	A
RAV0	USA	2019	MF817822.1	E
AF229	USA	2010	MF817821.1	E
AF227	USA	2019	MF817820.1	E
ALV-K-env-J	China	2019	MK638922.1	K
DL00766	China	2018	MH454773.1	B
WD16085b	China	2016	MG812188.1	B
ev21	China	2015	KY235336.1	A
GX14DJ3-18	China	2014	MH213216.1	A
SDAUAK-10	China	2016	KY767731.1	A
JS14CZ02	China	2014	KY490696.1	J
CH/JXTH2302	China	2023	OR670969.1	K
CH/JXTH2301	China	2023	OR670968.1	K
CH/JXTH2202	China	2023	OR670967.1	K
CH/JXTH2201	China	2023	OR670966.1	K
CH/JXTH2102	China	2023	OR670965.1	K
CH/JXND2101	China	2023	OR670962.1	K
CH/JXTH2101	China	2023	OR670961.1	K
CH/JXDX2302	China	2023	OR670960.1	K
CH/JXDX2202	China	2023	OR670958.1	K
CH/JXDX2201	China	2023	OR670957.1	K
CH/JXCR2101	China	2023	OR670952.1	K
GX14HG04	China	2016	KX058878.1	J
GX14LT07	China	2014	KX034517.1	J
GD1407	China	2014	KU500034.1	J
GX14FF03	China	2014	KU923579.1	B
SDAU1005	China	2011	KT156668.1	J

KmN_119	Japan	2022	OP644788.1	C
ALVDL21	China	2023	OQ749505.1	D
GX14YL03	China	2014	KR025484.1	J
GDFX0601	China	2014	KP686142.1	E
HLJE2020	China	2020	OK216743.1	K
LJAE0201	China	2020	MZ727181.1	E
HLJA0202	China	2018	MW804649.1	B
DPRJ21	India	2020	OK507207.1	J
ALV/Belgium/4439_001/2020	Belgium	2020	MZ367376.1	D

TABLE S2 The CIAV reference strains used in this study

Strain	Area	Date	Accession No.
98D02152	USA	2003	AF311892.2
CAV-EG-13	Egypt	2017	MH001560.1
HLJ15165	China	2015	KY486141.1
08AQ017A	South Korea	2020	MW091346.1
JZ2114	China	2021	OQ850281.1
JZ2113	China	2021	OQ850280.1
JZ2112	China	2021	OQ850279.1
JZ2110	China	2021	OQ850277.1
JZ2108	China	2021	OQ850275.1
HB2101	China	2021	OQ869196.1
Ahui1998-45	China	2020	OM799939.1
Ahhui1998	China	2018	OM799843.1
SD2015	China	2020	OL448846.1
EB8K	Turkey	2016	MT259317.1
KB1K	Turkey	2015	MT259306.1
AH4	China	2005	DQ124936.1
LF4	China	2005	AY839944.2
Harbin	China	2002	AF475908.1
CIA-1	USA	1999	L14767.1
JZ2115	China	2021	OQ850282.1
JZ2109	China	2021	OQ850276.1
Anhui1998-64	China	2020	OM799940.1
13-SD201907	China	2019	OQ116674.1
20-SD201911	China	2019	OQ116673.1
16-HN201912	China	2019	OQ116672.1
12-GD201810	China	2019	OQ116671.1
11-SD201907	China	2019	OQ116670.1
6-GD201810	China	2019	OQ116668.1
2-IM201808	China	2019	OQ116667.1
17-ZJ201912	China	2019	OQ116666.1
3-HN201912	China	2019	OQ116665.1
9-SD201904	China	2019	OQ116661.1
18-SD201909	China	2019	OQ116659.1
10-AH201911	China	2019	OQ116658.1
8-AH201911	China	2019	OQ116657.1
4-AH201911	China	2019	OQ116655.1
GX21122	China	2021	OQ267595.1
SD21731	China	2021	OP038388.1
JX21514	China	2021	OP038384.1
HuN21923	China	2021	OP038378.1
HuN21711	China	2021	OP038376.1

HuN21614	China	2021	OP038375.1
HuB21325	China	2021	OP038370.1
Hub21121	China	2021	OP038369.1
HeN21417	China	2021	OP038363.1
HaN21B31	China	2021	OP038358.1
GZ21213	China	2021	OP038348.1

TABLE S3 The ARV reference strains used in this study

Strain	Area	Date	Accession No.
MS01	China	2013	KY860639.1
S1133	USA	2023	OR612116.1
ARV_141045	USA	2021	OR612106.1
SD10-1	China	2010	KP288860.1
SD09-1	China	2009	KP288850.1
LN09-1	China	2009	KP288840.1
HB10-1	China	2010	KP288830.1
GX/2010/1	China	2010	KJ476702.1
S1133	China	2013	KF741769.1
S1133	China	2013	KF741759.1
C78	China	2013	KF741719.1
GuangxiR2	China	2000	KF741729.1
GX110058	China	2011	KF741739.1
GX110116	China	2011	KF741749.1
GuangxiR1	China	2000	KC183751.1
C-98	China	2008	EU616740.1
T-98	China	2008	EU616736.1
2408	Taiwan, China	2004	AY639613.1
1733	Taiwan, China	2004	AY639612.1
S1133	Taiwan, China	2004	AY639610.1
176	Canada	2004	AY557189.1
S1133	USA	2005	DQ300176.1
526	China	2013	KF741699.1
138	Canada	2004	AY557188.1
918	Taiwan, China	2004	AY639617.1
Reo/NC/Broiler/R1837308/18	USA	2018	OR546354.1
Turkey/USA/IA/2013/TARV-MN10	USA	2013	KJ874315.1
Turkey/USA/MN/2013/TARV-MN9	USA	2013	KJ874314.1
Turkey/USA/MN/2012/TERV-MN8	USA	2012	KJ874326.1
Turkey/USA/MN/2012/TERV-MN7	USA	2012	KJ874325.1
Turkey/USA/MN/2011/TERV-MN6	USA	2011	KJ874324.1
Turkey/USA/MN/2011/TERV-MN4	USA	2011	KJ874322.1
T1502036	USA	2015	MK616646.1
K1502030	USA	2015	MK583324.1
Reo/PA/Layer/01224A/14	USA	2014	KT428301.1
Reo/PA/Layer/01224A/14	USA	2014	AY639620.1
R2	Taiwan, China	2006	AY639619.1
1017-1	Taiwan, China	2004	AY639611.1
Reo/NC/Broiler/R1838167/18	USA	2018	OR546364.1
Reo/PA/Turkey/22342/13	USA	2013	KP173686.2
Turkey/USA/SD/2012/TARV-MN7	USA	2012	KJ874312.1

Turkey/USA/MN/2011/TARV-Crestview	USA	2011	KJ874304.1
K738/14	South Korea	2014	MF686698.1
Reo/PA/Broiler/05682/12	USA	2012	KM877328.1
Turkey/USA/MN/2011/TARV-MN1	USA	2011	KJ874306.1
SDYT2020	China	2020	MW394459.1
916SI	Taiwan, China	2004	AY639616.1

TABLE S4 The FAdV reference strains used in this study

Strain	Area	Date	Accession No.	Subtype
MSL/29/19	Indonesia	2019	MT104456.1	E8a
MSL/151/18	Indonesia	2019	MT104455.1	E8a
MSL/149/18	Indonesia	2019	MT104454.1	E8a
TR/BVKE/R/Y	Turkey	2019	MK937076.1	E8b
TR/BVKE/R/D-1	Turkey	2019	MK937075.1	E8b
TR/BVKE/CA/CYG	Turkey	2019	MK937074.1	E8b
TR/BVKE/CA/CAK	Turkey	2019	MK937073.1	E8b
TR/BVKE/R/B-8	Turkey	2019	MK937071.1	E8b
FAV-LS-170123	China	2017	MG547388.1	C10
SD14-1	China	2014	MF614119.1	E8b
ID-HCI-038	Peru	2016	MG765468.1	E8b
ID-HCI-023	Peru	2016	MG765463.1	E8b
ID-HCI-021	Peru	2015	MG765461.1	E8b
CH/CQBS/1504	China	2015	MF055634.1	D2
SD16-113	China	2016	KY426987.1	E8b
SD16-116	China	2016	KY426984.1	E8b
FAV-SDWF-140614-B	China	2014	KU981150.1	D3
FAV-JL-130131-B	China	2013	KU981148.1	D3
CHN-SX-FAdV-8a	China	2022	OR824927.1	E8a
H14.2	Uganda	2021	OP256899.1	E8a
H14.2	Uganda	2021	OP256897.1	E8a
H14.2	Uganda	2021	OP256896.1	E8a
H14.2	Uganda	2021	OP256894.1	E8a
H14.2	Uganda	2021	OP256893.1	E8a
SDLC210111	China	2021	ON959146.1	E8b
SDLY-22071601	China	2022	OP920979.1	D9
SDJN-22030201	China	2022	OP920978.1	D9
SDJN-22060901	China	2022	OP917910.1	E6
SDZB-21102601	China	2021	OP917909.1	E6
SDTA-21092901	China	2021	OP917908.1	E6
SDLY-21090401	China	2021	OP917907.1	E6
SDLY-21072501	China	2021	OP917906.1	E6
SDTA-21071301	China	2021	OP917905.1	E6
SDTA-21061902	China	2021	OP917904.1	E6
SDWF-21040701	China	2021	OP917903.1	E6
SDTA-21022602	China	2021	OP917902.1	E6
SDTA-21010702	China	2021	OP917901.1	E6
SDHZ-21010601	China	2021	OP917900.1	E6
CH/HBXT/1907	China	2019	MZ435840.1	E7
A-M-D	Iraq	2022	LC727624.1	B5
ID.MSL.485.21	Indonesia	2021	OK236347.1	E7

ID.MSL.425.21	Indonesia	2021	OK236345.1	E7
HBHD2021-H	China	2021	MW735942.1	D9
ISR/4346/2021	Israel	2021	MZ368700.1	E7
FAdV/chicken/China/SC/GA	China	2019	MT883493.1	E7
764	Canada	2009	JN112373.1	C10
SA84-08	South Africa	2008	HQ117911.1	E7
ATCC VR-830	Belgium	2001	AF339919.1	B5
CR119	Japan	2018	NC_038332.1	E6
CR119	Japan	2016	KT862808.1	E6
04-60057-902	Canada	2004	EF685478.1	D11
04-60057-923	Canada	2007	EF685466.1	D11
